# Association Between Gestational Diabetes Mellitus and Risk of Overall and Site-Specific Cancers (Pancreatic, Liver, Thyroid, Lung): A Systematic Review and Meta-Analysis

**DOI:** 10.3390/life15050808

**Published:** 2025-05-19

**Authors:** Lv Tian, Yixuan Wen, Chuanwang Liu, Tao Li, Jun Fan

**Affiliations:** 1 Institute of Fundamental and Frontier Sciences, University of Electronic Science and Technology of China, Chengdu 611731, China; 2 Shimmer Center, Tianfu Jiangxi Laboratory, Chengdu 641419, China

**Keywords:** meta-analysis, gestational diabetes mellitus, overall cancer, pancreatic cancer, thyroid cancer

## Abstract

**Background**: Gestational diabetes mellitus (GDM) is a common endocrine and metabolic disorder during pregnancy. However, current studies have not reached a consensus on the correlation between GDM and the risk of developing cancers. **Objective**: This systematic review and meta-analysis aims to comprehensively evaluate the association between GDM and the risk of overall cancer and cancers at specific sites (pancreatic cancer, thyroid cancer, liver cancer, lung cancer). **Methods**: A systematic search was conducted in PubMed, Web of Science, Scopus, EMBASE, and Cochrane Library databases from the establishment of the databases to 16 January 2025. Two researchers independently assessed the quality of the included studies using the Newcastle-Ottawa Scale and extracted relevant data. Data were analyzed using STATA Version 17.0. **Results:** This systematic review and meta-analysis included a total of 8 studies involving 1,936,836 participants. We calculated the pooled hazard ratio (HR) to evaluate the association, and the results showed that the pooled HR for overall cancer risk was 1.16 (95%CI: 1.04–1.28), indicating a significant increase in the risk of overall malignancies among patients with GDM. GDM was also significantly associated with the risk of pancreatic cancer (HR = 2.80; 95%CI: 1.20–6.55), thyroid cancer (HR = 1.21; 95%CI: 1.08–1.36), and liver cancer (HR = 1.33; 95%CI: 1.10–1.61). Additionally, the association between GDM and lung cancer was close to being statistically significant (HR = 1.19; 95%CI: 0.98–1.44). **Conclusion:** Our study suggests that GDM is associated with an increased risk of overall cancer, as well as pancreatic cancer, thyroid cancer, and liver cancer.

## 1. Introduction

Gestational diabetes mellitus (GDM) refers to the onset or first detection of glucose intolerance during pregnancy [1]. During pregnancy, women frequently develop progressive insulin resistance, driven by rising maternal obesity rates and placental hormone-induced insulin desensitization [2]. Although many pregnant women can offset insulin resistance through heightened insulin secretion, patients with GDM are unable to effectively compensate due to impaired insulin secretory function, leading to a state of hyperglycemia and potentially unveiling underlying chronic metabolic abnormalities. As a result, GDM stands as a commonly encountered metabolic dysfunction that augments complication risks in both mother and baby during gestation [1]. Multiple studies have demonstrated that the prevalence of GDM is continuously rising, influenced by global increases in obesity rates, sedentary lifestyles, and unhealthy dietary habits [3,4]. The global prevalence of GDM is approximately 15.6 percent [5].

The primary risk factors for GDM include polycystic ovary syndrome, obesity or overweight status of the mother, a family history of type 2 diabetes mellitus (T2DM), prediabetes, a history of previous fetal death, and advanced maternal age at pregnancy [1]. In addition, environmental factors also play a role. For instance, exposure to certain environmental chemical substances, such as bisphenol A, commonly found in plastics, may interfere with normal metabolic processes, increasing the risk of GDM [6]. From a genetic perspective, genetic susceptibility is a crucial factor in the onset of GDM [7]. Polymorphisms in genes such as KCNQ1 and GCK are associated with increased susceptibility to GDM [8,9]. The polymorphism of the KCNQ1 gene may affect potassium channel function, which is related to the regulation of insulin secretion [7]. Mutations in the GCK gene can lead to abnormal glucokinase activity, disrupting glucose sensing and insulin secretion in pancreatic β-cells [10]. From a pathophysiological perspective, the mechanism of GDM is relatively complex: During normal pregnancy, physiological insulin resistance occurs in pregnant women due to factors such as placental hormone secretion, enhanced inflammatory response, and excessive lipolysis [11]. This insulin resistance primarily affects the liver, muscle, and adipose tissue, resulting in a significant reduction in their sensitivity to insulin [12]. Additionally, patients with GDM have defective pancreatic beta-cell function, unable to secrete sufficient insulin to compensate for insulin resistance [13]. Therefore, the typical characteristics of GDM are hyperglycemia, insulin resistance, and hyperinsulinemia [14,15], and these metabolic abnormalities are closely associated with uncontrolled cell proliferation and an increased risk of cancer development [14,16,17].

Cancer remains a significant disease that seriously threatens human health [18]. According to data from the World Health Organization, both the global incidence and mortality rates of cancer have been on the rise in recent years. Specifically, pancreatic cancer, characterized by its high malignancy and poor prognosis, has shown an increasing trend in incidence [18,19]. Liver cancer, which is prevalent in areas with a high incidence of hepatitis, is often diagnosed in the intermediate or advanced stages [18,20]. Thyroid cancer has experienced a rapid growth in incidence in recent years [18,19], while lung cancer remains one of the tumors with the highest incidence and mortality rates [18,20]. The escalating burden of cancer not only consumes considerable medical resources but also imposes a substantial impact on the quality of life of patients and their families [18].

In recent years, numerous studies have explored the association between GDM and cancer risk. However, the findings of these studies have been inconsistent. Regarding the risk of overall cancer, several studies [21,22,23] suggest that GDM increases the risk of overall cancer, while other researches [24,25] hold different views. Given the inconsistencies in research findings, the global rise in GDM prevalence, and the close relevance of GDM to public health and clinical practice, we conducted a meta-analysis to evaluate the existing evidence comprehensively and systematically on the association between GDM and the risk of overall cancer, as well as pancreatic cancer, thyroid cancer, liver cancer, and lung cancer.

## 2. Methods

### 2.1. Registration Information

This study adhered to the Preferred Reporting Items for Systematic Reviews and Meta-Analyses (PRISMA) guidelines [26]. Additionally, it was prospectively registered with the International Prospective Registry of Systematic Reviews under the identification number CRD420251012546.

### 2.2. Search Strategy

A comprehensive search for original studies related to the association between GDM and cancer was conducted using PubMed, Embase, Scopus, the Cochrane Library, and Web of Science. The search covered the period from the inception of each database up to 16 January 2025. Search terms consisted of a combination of subject headings and free words. The search strategy for PubMed was shown below: ((“Diabetes, Gestational”[Mesh]) OR (((Gestational Diabetes Mellitus) OR (GDM)) OR (Diabetes, Pregnancy-Induced))) AND ((“Neoplasms”[Mesh]) OR (((Cancer*) OR (Tumor*)) OR (Carcinoma*))). It was adapted for the other databases, with terminology adjusted according to the specific syntax and indexing system of each database (Table S1).

### 2.3. Eligibility Criteria

The literature search was limited to published English articles, and eligible studies must meet the following criteria:

Population (P): Women with Gestational Diabetes Mellitus.

Exposure: The exposure factor was GDM. The study should focus on the association between GDM and cancer risk.

Comparison (C): Women without Gestational Diabetes Mellitus.

Outcome (O): The outcome measures should include risk values for overall cancer as well as pancreatic cancer, thyroid cancer, liver cancer, and lung cancer. The study should report odds ratios (OR), relative risks (RR), or hazard ratios (HR) with their corresponding 95% confidence intervals (CI) or provide sufficient data to calculate the effect size between GDM and cancer.

Study design (S): The study design should be a cohort study or a case-control study.

The exclusion criteria are as follows:

(1) Duplicate literature.
(2) Review articles or meta-analyses.
(3) Protocols or guidelines.
(4) Conference abstracts or letters.
(5) Animal studies.
(6) Mechanism studies.
(7) Irrelevant studies.
(8) Abstract only, full text not available.

### 2.4. Study Selection

The search results from various databases were imported into EndNote X9 software for deduplication and the literature management. To ensure the accuracy and objectivity of the data, two independent reviewers (LT and YXW) conducted an initial screening of the titles and abstracts of the retrieved literature according to pre-set inclusion criteria. For studies that passed the initial screening, the full texts were obtained and further screened to determine the final included studies. During the screening process, any disagreements between the two reviewers were resolved through discussion to reach a consensus; if necessary, a third reviewer (JF) participated in the discussion and provided an arbitral opinion.

### 2.5. Data Extraction

This study strictly followed the PRISMA statement for data extraction to ensure the systematicity of the research methodology. Two reviewers (LT and YXW) independently extracted data using a pre-established data extraction form, while a third author (JF) cross-checked the accuracy of the results. The extracted data included author (year), country, study design, age (GDM/non-GDM), sample size, cancers, RR (95% CI), HR (95% CI), follow-up time (years), quality (scores), and adjustment factors.

### 2.6. Quality Assessment

The Newcastle-Ottawa Quality Assessment Scale is a tool designed to evaluate the quality of both cohort and case-control studies, allowing for the assessment of the quality of each study [27]. It consists of 8 items grouped into three dimensions: selection of the groups, comparability of the groups, and exposure/outcome. The maximum score on this scale is 9 points. Studies scoring below 4 points are considered low quality, those scoring between 4 and 6 points are of moderate quality, and those scoring between 7 and 9 points are deemed high quality [27].

### 2.7. Data Synthesis and Analysis

To evaluate the association between GDM and cancer, we synthesized hazard ratios (HRs) and their corresponding 95% confidence intervals (CIs). Given the relatively low relative risk (RR) of cancer in relation to GDM, we anticipated that RR would yield similar estimates to HR; therefore, all RRs were considered equivalent to HRs for the pooled analysis. Initially, we employed the Q test to assess inter-study heterogeneity, with a significance level set at *p* = 0.1. Subsequently, we determined the degree of heterogeneity based on the I^2^ statistic: if I^2^ < 50%, indicating non-significant heterogeneity, a fixed-effects model was utilized; if I^2^ ≥ 50%, suggesting significant statistical heterogeneity [28], a random-effects model was chosen. In addition, we also conducted the following analyses: performing a sensitivity analysis using the leave-one-out method [29], evaluating publication bias by observing the symmetry of the funnel plot, and calculating Begg’s test value and Egger’s test value [30,31]. All statistical analyses were performed using Stata 17.0, with statistical significance defined as *p* < 0.05.

## 3. Results

### 3.1. Compliance with the Registered Protocol

There were no inconsistencies with the pre-registration protocol.

### 3.2. Study Selection

**Figure 1:**
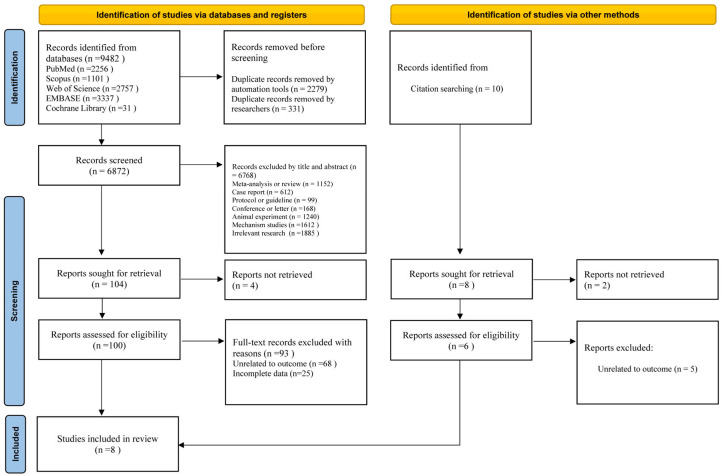
PRISMA flow chart for study selection.

The selection process and reasons for exclusion in this study are illustrated in Figure 1. We retrieved 9482 articles from five databases: PubMed, Embase, Scopus, Cochrane Library, and Web of Science. After removing duplicates using automated tools and manual screening based on titles and abstracts, we selected 104 articles for further evaluation. Among them, four studies were excluded due to the inability to access the full text, leaving 100 studies for full-text evaluation. Following the full-text assessment, 93 studies were excluded because they were not relevant to the required outcome measures, or complete data were not available. Ultimately, seven studies met the inclusion criteria. Additionally, we conducted a related citation-tracking search and identified ten additional studies. After applying the inclusion and exclusion criteria, one more study was included. Finally, we obtained eight studies [21,22,23,24,25,32,33,34] for meta-analysis.

### 3.3. Study Characteristics

This meta-analysis included eight cohort studies published between 2007 and 2024, originating from Canada, Israel, South Korea, and Taiwan. The sample sizes of these cohort studies ranged from 37,926 to 990,572, covering a broad spectrum of ages among both individuals with GDM and non-GDM. The included studies focused on the associations between GDM and the risks of overall cancer, pancreatic cancer, liver cancer, thyroid cancer, or lung cancer (associations between GDM and the risk of one or more types of cancer), reporting RR and HR with their corresponding 95% CIs. Follow-up durations varied from 5.19 years (standard deviation of 3.9) to 13.1 years (standard deviation of 5.2), with some studies utilizing median or mean follow-up times. All studies were of high quality, scoring between 7 and 8 (Table S2). The included studies controlled for confounders: each adjusted for different confounders, such as age, number of births, socioeconomic factors, smoking status, and body mass index (BMI). More information about the main results of each study is shown in Table 1.

### 3.4. Outcomes of the Meta-Analysis

**Figure 2:**
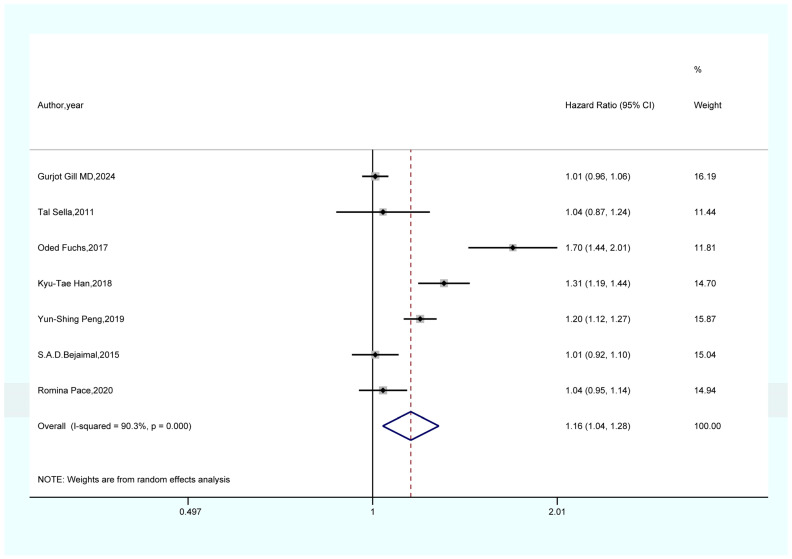
Forest plot of the association between GDM and overall cancer [21,22,23,24,25,32,34].

**Figure 3:**
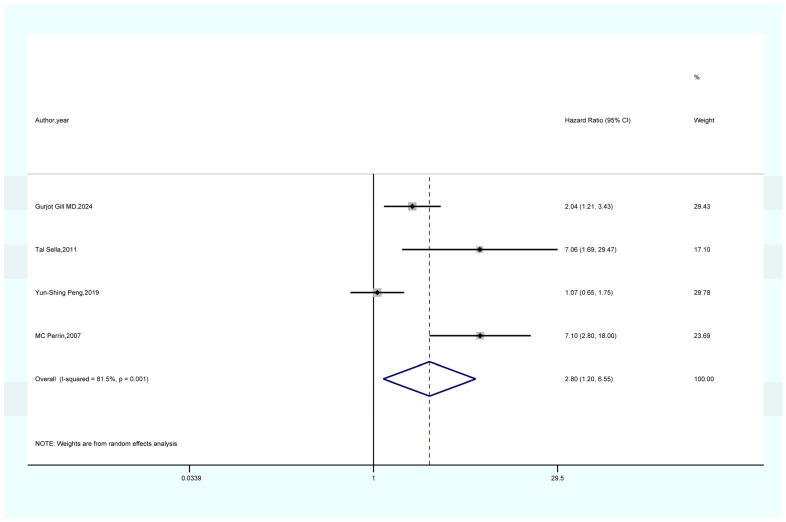
Forest plot of the association between GDM and pancreatic cancer [23,24,33,34].

**Figure 4:**
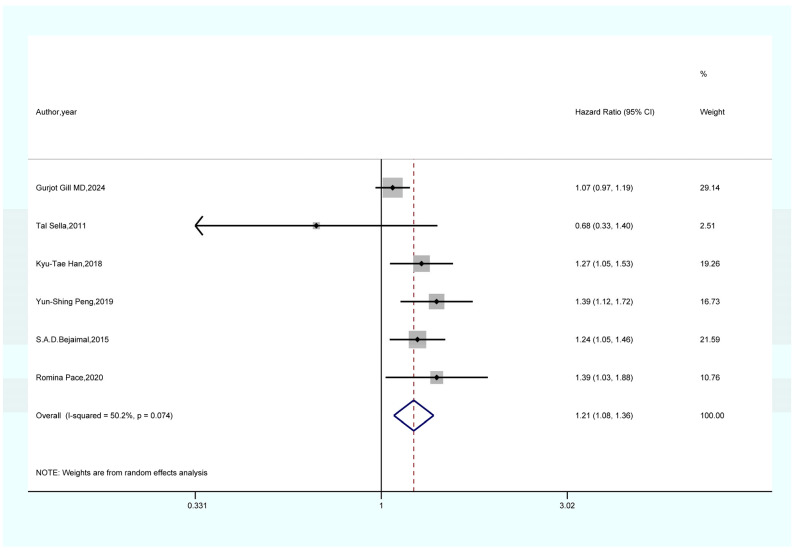
Forest plot of the association between GDM and thyroid cancer [22,23,24,25,32,34].

**Figure 5:**
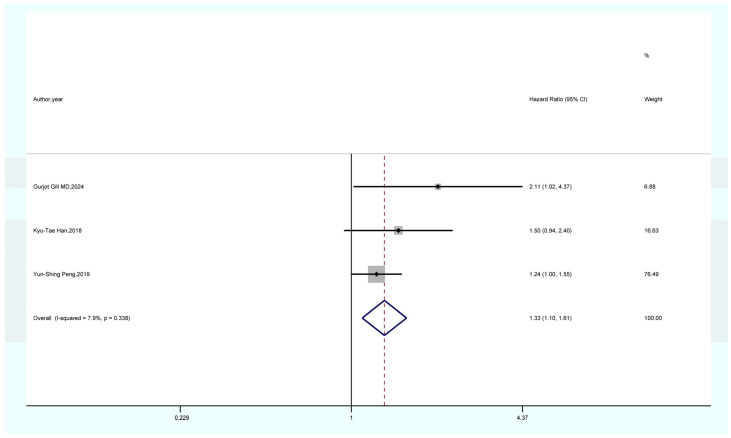
Forest plot of the association between GDM and liver cancer [22,23,24].

As shown in Figure 2, the results of a meta-analysis encompassing seven studies suggest that women with a prior history of GDM face a notably elevated risk of developing overall cancer when compared to those without GDM (HR = 1.16, 95% CI = 1.04–1.28, I^2^ = 90.3). As shown in Figure 3, a meta-analysis of four studies showed that GDM was significantly associated with pancreatic cancer risk (HR = 2.80, 95% CI = 1.20–6.55, I^2^ = 81.5%). As demonstrated in Figure 4, the findings from a meta-analysis of six studies reveal a significant correlation between GDM and the risk of thyroid cancer (HR = 1.21, 95% CI = 1.08–1.36, I^2^ = 50.2). The findings of a meta-analysis (Figure 5) involving three studies imply that females with a history of GDM are at a markedly heightened risk of developing liver cancer in comparison to those without GDM (HR = 1.33, 95% CI = 1.10–1.61, I^2^ = 7.9%). As illustrated in supplementary material (Figure S1), the findings from the meta-analysis of three studies suggest that there is no association between GDM and the risk of lung cancer (HR = 1.19, 95% CI = 0.98–1.44, I^2^ = 19.3).

### 3.5. Sensitivity Analysis

As shown in Figure S2, the sensitivity analysis results for overall cancer, pancreatic cancer, and thyroid cancer demonstrate that the findings from the synthesis analysis remain robust even after excluding any individual study.

### 3.6. Publication Bias

Although the results of the funnel plot (Figure S3) show an asymmetry between the studies, as shown in Table S3, the results of Begg’s test (Z = 1.50, *p* = 0.333) and Egger’s test (T = 1.25, *p* = 0.265) for overall cancer, the Begg’s test (Z = 1.02, *p* = 0.308) and Egger’s test (T = 2.24, *p* = 0.154) for pancreatic cancer, and the Begg’s test (Z = 0.75, *p* = 0.452) and Egger’s test (T = 0.52, *p* = 0.628) for thyroid cancer indicated that there is no significant publication bias in the pooled results of the meta-analysis.

## 4. Discussion

GDM is the most common metabolic disease, with a global prevalence of 15.6 percent [5]. Meanwhile, cancer poses a significant threat to human health, with persistently high global incidence and mortality rates [35]. Both GDM and cancer are public health issues that have garnered considerable attention. However, despite the widespread focus on their relationship, existing conclusions remain controversial [22,23,24,25]. For example, a systematic review has indicated that the risk of GDM and gynaecological cancers as a whole remains controversial, and GDM is also associated with the risk of developing certain solid tumours in offspring [36]. Studies by Fuchs et al. [21], Han et al. [22], and Peng et al. [23] suggest that GDM increases the overall risk of cancer development, while Gill [24] and Romina Pace et al. [25] hold different views. In addition, various studies have drawn different conclusions regarding the relationship between GDM and the risk of various specific types of cancer. Gill’s [24] research indicates that GDM elevates the risk of pancreatic cancer, whereas Peng et al. [23] believe there is no association between the two; Bejaimal [32] points out a significant correlation between GDM and thyroid cancer, but Sella et al. [34] draw contradictory conclusions; Peng et al. [23] argue that GDM is significantly associated with lung cancer risk, whereas Pace et al. [25] disagree; Gill et al. [24] note an increased risk of liver cancer among women with a history of GDM, whereas Han et al. [22] suggest no association between GDM and liver cancer risk.

Based on these inconsistent conclusions, this study conducted a meta-analysis of eight existing studies. This systematic review and meta-analysis comprehensively evaluated the relationship between GDM and the risk of overall cancer as well as cancers of specific sites (pancreatic cancer, thyroid cancer, liver cancer, and lung cancer). The results indicated that GDM is significantly associated with an increased risk of overall cancer, pancreatic cancer, thyroid cancer, and liver cancer, and the association with lung cancer is close to statistical significance. Begg’s and Egger’s tests for overall cancer, pancreatic cancer, thyroid cancer, liver cancer, and lung cancer showed no significant publication bias.

### 4.1. Potential Mechanisms of GDM and Cancer

GDM is a specific glucose metabolism disorder during pregnancy. Our findings of this study indicate that GDM is associated with a significantly increased risk of overall cancer, pancreatic cancer, thyroid cancer, and liver cancer, suggesting a complex mechanism of action for GDM in the development and progression of these cancers.

#### 4.1.1. GDM and Overall Cancer

Our meta-analysis showed that GDM was significantly associated with overall cancer risk (HR = 1.16, 95% CI = 1.04–1.28). This finding is consistent with the theory that metabolic disorders can lead to cancer. Patients with GDM are chronically exposed to hyperglycemia, which significantly exacerbates insulin resistance [37,38]. This insulin resistance triggers a compensatory increase in insulin secretion, disrupting the insulin-like growth factor (IGF) system. Under normal conditions, insulin interacts with IGF-1 receptors. However, when insulin levels rise, this pathway becomes overactivated, increasing the phosphorylation of serine and threonine residues on IGFBPs [39]. Following the phosphorylation of IGFBPs, their affinity for IGF-1 decreases, resulting in the release of IGF-1 that was originally tightly bound to IGFBPs, causing a relative increase in the level of free IGF-1 in the blood [40]. IGF-1 exerts dual effects by activating downstream signaling pathways, including phosphatidylinositol-3 kinase (PI3K)/protein kinase B (Akt) and mitogen-activated protein kinase (MAPK) [41]. On one hand, it enhances the expression of cyclin D1, promoting the transition of cells from the G1 to S phase and accelerating cell proliferation. On the other hand, it suppresses the expression of pro-apoptotic proteins such as Bax while activating anti-apoptotic proteins such as Bcl-2, thereby inhibiting cell apoptosis and creating a favorable environment for the growth and survival of tumor cells [42,43,44,45,46]. Concurrently, in a hyperglycemic environment, glucose auto-oxidation, activation of the polyol pathway, and hyperactivation of the protein kinase C (PKC) pathway contribute to the generation of reactive oxygen species (ROS) [42,47]. Excessive ROS attacks DNA, causing damage such as base modifications and strand breaks [48,49,50]. Furthermore, ROS activates transcription factors such as nuclear factor-κB (NF-κB) through oxidative stress, inducing the expression of inflammatory cytokines such as tumor necrosis factor-α (TNF-α) and interleukin-6 (IL-6). This further disrupts intracellular signaling, compromises cellular genomic stability, and increases the risk of cancer development [48,49,50].

#### 4.1.2. GDM and Pancreatic Cancer

In pancreatic cancer (HR = 2.80, 95% CI = 1.20–6.55), hyperinsulinemia in patients with GDM may play a significant role. Insulin can directly bind to insulin receptors on the surface of pancreatic duct epithelial cells, activating the PI3K/Akt and MAPK signaling pathways and stimulating abnormal cell proliferation [51,52]. Additionally, insulin can upregulate the expression of cyclooxygenase-2 (COX-2), promoting the synthesis of prostaglandin E2 (PGE2). PGE2, in turn, enhances cell proliferation and migration capabilities [51,53,54]. Furthermore, under hyperglycemic conditions, glucose metabolites such as methylglyoxal (MGO) accumulate. MGO can form advanced glycation end products (AGEs) with biological macromolecules such as proteins and nucleic acids [55,56]. The binding of AGEs to their receptor (RAGE) activates the NF-κB signaling pathway, leading to the release of inflammatory factors and simultaneous activation of the MAPK signaling pathway, inducing malignant cell transformation [55,56]. The chronic low-grade inflammatory microenvironment triggered by GDM is characterized by macrophage infiltration and continuous release of inflammatory factors [55,56]. These inflammatory factors, such as TNF-α and IL-6, can upregulate the expression of matrix metalloproteinases (MMPs), degrading the extracellular matrix and promoting the invasion and metastasis of pancreatic cancer cells [55,56].

#### 4.1.3. GDM and Thyroid Cancer

There is a significant association between GDM and the risk of thyroid cancer (HR = 1.21, 95% CI = 1.08–1.36), and this association is likely mediated by the hormonal changes induced by GDM, which have a significant impact on thyroid carcinogenesis. In the GDM state, the level of serum thyroid hormone binding globulin (TBG) may be altered due to glycosylation, affecting its binding capacity with thyroid hormones [57], thereby disrupting the normal metabolism and signal transduction of thyroid hormones [57]. Thyroid hormones are crucial for maintaining the normal function, proliferation, and differentiation of thyroid cells, and abnormalities in their signaling pathways may prompt abnormal proliferation of thyroid cells [57]. Meanwhile, the immune dysfunction associated with GDM cannot be ignored. A hyperglycemic environment inhibits T lymphocyte function, reduces the activity of cytotoxic T lymphocytes (CTL) and natural killer cells (NK), and weakens the body’s immune surveillance and killing ability against thyroid cancer cells [58,59]. Additionally, increased inflammatory factors such as TNF-α and IL-6 can alter the local immune microenvironment of the thyroid, inhibiting immune cells from recognizing and attacking tumor cells, making it easier for tumor cells to develop and progress [60,61].

#### 4.1.4. GDM and Liver Cancer

Our study showed that GDM significantly increased the risk of liver cancer (HR = 1.33, 95% CI = 1.10–1.61). In the context of liver cancer, GDM significantly disrupts glucose metabolism in the liver. Hyperglycemia stimulates increased fatty acid synthesis in the liver. At the same time, insulin resistance inhibits fatty acid β-oxidation, leading to excessive fat deposition in the liver and potentially progressing to non-alcoholic fatty liver disease (NAFLD) [62,63]. During the progression of NAFLD, steatotic hepatocytes undergo oxidative stress and endoplasmic reticulum stress, activating the c-Jun N-terminal kinase (JNK) signaling pathway [62,64,65]. JNK phosphorylates and activates the transcription factor c-Jun, upregulating the expression of inflammatory cytokines, promoting hepatic stellate cell activation, and ultimately leading to liver fibrosis [62,64,65]. Persistent fibrosis can further evolve into cirrhosis, ultimately increasing the risk of liver cancer [62]. From a molecular perspective, insulin resistance leads to hyperactivation of the PI3K/Akt/mTOR signaling pathway in the liver [62,65,66,67]. As a key regulator of cell growth and metabolism, mTOR promotes protein and lipid synthesis while inhibiting autophagy, providing the energetic and material basis for the proliferation and survival of hepatoma cells [62,65,66,67].

Although we have explored the potential impact of GDM on cancer development, existing research has not fully elucidated the specific mechanisms linking GDM to the progression of specific cancer types. Therefore, further basic and clinical studies are needed to clarify the relationship between GDM and cancer.

### 4.2. Comparison with the Published Systematic Review and Meta-Analysis

A previous systematic review has explored the correlation between GDM and cancer risk [36]. We compared our study with this systematic review: in terms of the literature search, Slouha et al. conducted their literature search up to 1 December 2023, whereas our research extended the search deadline to 16 January 2025, incorporating more recent literature. Methodologically and in terms of results, Slouha et al. employed a narrative synthesis approach to report their findings. Conversely, our study adopted a more rigorous methodology, performing a meta-analysis on outcome measures to present our results. Additionally, we verified the robustness of our findings through sensitivity analysis and publication bias assessment. Consequently, the conclusions drawn from our research carry greater persuasiveness.

### 4.3. Limitations and Strengths

Our meta-analysis has the following limitations:

(1) Most of the included studies were observational, which may be subject to confounding factors and biases.
(2) Variations in the definitions and diagnosis codes of GDM and cancer across different studies could potentially affect the accuracy of the results.
(3) The limited number of studies meeting the criteria and included in the analysis hindered the possibility of conducting subgroup analyses based on study characteristics such as age, region, and follow-up time, thereby impeding the exploration of sources of heterogeneity.
(4) The included studies differed in terms of the types and numbers of cancers investigated, with some covering multiple cancers and others focusing on a single type. Due to the limited number of studies, sensitivity analysis and publication bias testing could not be performed for liver and lung cancer data, requiring caution in interpreting the results. Future studies with larger sample sizes are needed for further validation.

Despite the aforementioned limitations, this meta-analysis still possesses several notable strengths:

(1) The results of the sensitivity analysis demonstrate the robustness and reliability of our primary findings, including the associations between GDM and overall cancer, pancreatic cancer, and thyroid cancer. Both Begg’s and Egger’s tests did not indicate any publication bias.
(2) This study represents the first meta-analysis to investigate the relationship between GDM and the risk of overall cancer, providing valuable guidance for clinical practice.
(3) Furthermore, this study explores the potential underlying mechanisms between GDM and cancer, offering a stronger theoretical foundation to support the research conclusions.

### 4.4. Clinical Implications

Our findings have profound clinical implications: (1) Women with a history of GDM should be considered as a high-risk group for cancer and are advised to undergo regular cancer screenings. (2) Long-term management for women who have had GDM should include lifestyle interventions and metabolic control to reduce the risk of future cancer. (3) Further research is needed to explore the underlying mechanisms between GDM and cancer and to develop targeted prevention and treatment strategies.

## 5. Conclusions

In summary, there is a significant association between GDM and the overall risk of cancer, as well as the incidence risks of pancreatic cancer, thyroid cancer, and liver cancer. This study suggests that GDM may be an important factor in predicting future cancer risks for women. Therefore, patients with GDM should be considered a high-risk population for cancer and advised to undergo regular cancer screenings and long-term metabolic management. In the future, large-scale, high-quality clinical studies and basic experiments are needed to further explore the underlying mechanisms between GDM and cancer, leading to the development of targeted prevention and treatment strategies.

## Figures and Tables

**Table 1:** Characteristics of individual studies included in the meta-analysis.

Author (Year)	Country	Study Design	Age (GDM/Non-GDM)	Sample Size	Main Outcome			Follow-Up Time (Years)	Quality (Scores)
					Cancers	RR (95% CI)	HR (95% CI)		
Gurjot Gill MD, 2024 [24]	Canada	Cohort study	33(IQR 33–37)	297,771	Overall Cancer; Pancreatic Cancer; Liver Cancer; Thyroid Cancer; Lung Cancer		1.01 (0.96, 1.06); 2.04 (1.21, 3.43); 2.11 (1.02, 4.38); 1.07 (0.97, 1.19); 0.98 (0.71, 1.34)	8 (IQR 4–13)	High (7)
Romina Pace, 2020 [25]	Canada	Cohort study	44.9% ≤ 30; 55.1% > 30	68,588	Overall Cancer; Thyroid Cancer; Lung Cancer		1.04 (0.95, 1.14); 1.39 (1.03, 1.89); 1.20 (0.73, 1.99)	13.1 (SD 5.2)	High (7)
Yun-Shing Peng, 2019 [23]	Taiwan, China	Cohort study	31.61 (SD 4.54)/28.83 (SD 4.89)	990,572	Overall Cancer; Pancreatic Cancer; Liver Cancer; Thyroid Cancer; Lung Cancer		1.197 (1.125, 1.274); 1.072 (0.655, 1.755); 1.242 (0.998, 1.545); 1.389 (1.121, 1.721); 1.372 (1.044, 1.803)	6.84 (SD 3.05)	High (7)
Kyu-Tae Han, 2018 [22]	South Korea	Cohort study	28.25 (SD 3.28)/27.28 (SD 3.02)	102,900	Overall Cancer; Liver Cancer; Thyroid Cancer		1.31 (1.192, 1.449); 1.5 (0.939, 2.397); 1.27 (1.054, 1.532)	10	High (8)
Oded Fuchs, 2017 [21]	Israel	Cohort study	31.8 (SD 5.9)/28.1 (SD 5.9)	104,715	Overall Cancer		1.7 (1.5, 2.1)	11.2 (Average)	High (7)
S.A.D. Bejaimal, 2015 [32]	Canada	Cohort study	32 (IQR 28–35)	149,049	Overall Cancer; Thyroid Cancer		1.01 (0.93, 1.11); 1.24 (1.05, 1.46)	8 (IQR 5–12)	High (7)
Tal Sella, 2011 [34]	Israel	Cohort study	32.74 (SD 5.51)/30.59 (SD 5.51)	185,315	Overall Cancer; Pancreatic Cancer; Thyroid Cancer		1.04 (0.87, 1.24); 7.06 (1.69, 29.45); 0.68 (0.33, 1.39)	5.19(SD 3.9)	High (8)
MC Perrin, 2007 [33]	Israel	Cohort study	>35	37,926	Pancreatic Cancer	7.1 (2.8, 18.0)		38.0 (Average)	High (7)

Notes: GDM, Gestational diabetes mellitus; RR: Relative Risk; HR: Hazard Ratio; CI: Confidence Interval; IQR: Interquartile Range; SD: Standard Deviation. GDM: Gestational Diabetes Mellitus; IQR: Interquartile Range; SD: Standard Deviation.

## Data Availability

All data are collected in a separate file available from the corresponding author.

## Supplementary Materials

The following supporting information can be downloaded at: https://www.mdpi.com/article/10.3390/life15050808/s1. Figure S1: Forest plot of the association between GDM and lung cancer; Figure S2: Sensitivity analyses; Figure S3: Funnel plot; Table S1: Search strategy of each database; Table S2: Newcastle-Ottawa Quality Assessment Scale—Quality Assessment of included studies; Table S3: Begg’s and Egger’s test values.
