# Supplementary material for: Association Between Gestational Diabetes Mellitus and Risk of Overall and Site-Specific Cancers (Pancreatic, Liver, Thyroid, Lung): A Systematic Review and Meta-Analysis
Source: Life (Basel). 2025 May 19;15(5):808. doi: 10.3390/life15050808 (PMC12113273; doi:10.3390/life15050808)
Supplement: Supplementary file 1 [file life-15-00808-s001.zip › Supplementary material.pdf]

Association Between Gestational Diabetes Mellitus and Risk of Overall and Site-Specific Cancers (Pancreatic, Liver, Thyroid, Lung): A Systematic Review and Meta-Analysis

Table S1 Search strategy of each database. 2
Table S2 Newcastle-Ottawa Quality Assessment Scale - Quality Assessment of included studies. 3
Figure S1 5
Figure S2 6
Figure S3 7
Table S3 Begg's and Egger's test values 8
Figure Legends: .... **Error! Bookmark not defined.**

Table S1 Search strategy of each database.

	**2. Web of science**	
#1	"Diabetes, Gestational" (Topic) or "Gestational Diabetes Mellitus" (Topic) or GDM (Topic) or "Diabetes, Pregnancy-Induced" (Topic)	37,444
#2	"Diabetes, Gestational" (Topic) or "Gestational Diabetes Mellitus" (Topic) or GDM (Topic) or "Diabetes, Pregnancy-Induced" (Topic)	8,494,988
#3	#1 AND #2	2757
	**3. Scopus**	
#1	(TITLE-ABS-KEY ("Diabetes, Gestational") OR TITLE-ABS-KEY ("Gestational Diabetes Mellitus") OR TITLE-ABS-KEY ("GDM") OR TITLE-ABS-KEY ("Diabetes, Pregnancy-Induced"))	28,747
#2	("Neoplasm*") OR TITLE-ABS-KEY ("Cancer*") OR TITLE-ABS-KEY ("Tumor*") OR TITLE-ABS-KEY ("Carcinoma*"))	6,597,582
#3	#1 AND #2	1101
	**4. Embase**	
#1	'diabetes, gestational'/exp OR 'diabetes, gestational' OR 'gestational diabetes mellitus':ti,ab,kw OR gdm:ti,ab,kw OR 'diabetes, pregnancy-induced':ti,ab,kw	61,725
#2	'neoplasms'/exp OR 'neoplasms' OR cancer*:ti,ab,kw OR tumor*:ti,ab,kw OR carcinoma*:ti,ab,kw	7,617,108
#3	#1 AND #2	3,337
	**5. Cochrane Library**	
	("Diabetes, Gestational"):ti,ab,kw OR ("Gestational Diabetes Mellitus"):ti,ab,kw OR (GDM):ti,ab,kw OR ("Diabetes, Pregnancy-Induced"):ti,ab,kw	279,782
	(Neoplasms):ti,ab,kw OR (Cancer*):ti,ab,kw OR (Tumor*):ti,ab,kw OR (Carcinoma*):ti,ab,kw	3136
	#2 and #3	31

Table S2 Newcastle-Ottawa Quality Assessment Scale - Quality Assessment of included studies.

Author (year)	Adjustment factors	Selection (Max 4 points)				Comparability (Max 2 points)	Outcome			Total Score (Max 9 points)
		Representativeness of the Exposed Cohort	Selection of the Non-Exposed Cohort	Ascertainment of Exposure	Demonstration That Outcome of Interest Was Not Present at Start of Study	Comparability of Cohorts on the Basis of the Design or Analysis	Assessment of Outcome	Was Follow-Up Long Enough for Outcomes to Occur	Adequacy of Follow Up of Cohorts	
Gurjot Gill MD, 2024	Adjusted[1]	1	1	1	0	2	1	1	0	High (7)
Romina Pace, 2020	Adjusted[2]	1	1	1	0	2	1	1	0	High (7)
Yun-Shing Peng, 2019	Adjusted[3]	1	1	1	0	2	1	1	0	High (7)
Kyu-Tae Han, 2018	Adjusted[4]	1	1	1	0	2	1	1	1	High (8)
Oded Fuchs, 2017	Adjusted[5]	1	1	1	0	2	1	1	0	High (7)
S.A.D.Bejaimal,2015	Adjusted[6]	1	1	1	0	2	1	1	0	High (7)
Tal Sella, 2011	Adjusted[7]	1	1	1	0	2	1	1	1	High (8)

MC Perrin, 2007	Adjusted[8]	1	1	1	0	2	1	1	0	High (7)

Notes: GDM, Gestational diabetes mellitus; RR: Relative Risk; HR: Hazard Ratio; CI: Confidence Interval; IQR: Interquartile Range; SD: Standard Deviation.

GDM: Gestational Diabetes Mellitus; IQR: Interquartile Range; SD: Standard Deviation.

Adjusted[1]: Age, parity, year of delivery, neighbourhood income quintile, urban vs rural residence, recent immigration status, surname - based ethnicity, number of core primary care visits in 3 years before delivery, endocrinologist visits in follow - up period.

Adjusted[2]: Gestational hypertension, preterm delivery, infant size, parity, prior comorbidity, material deprivation index, and ethnicity.

Adjusted[3]: Age, hypertension, dyslipidemia, liver disease, infertility and kidney disease.

Adjusted[4]: Maternal age, smoking, BMI before pregnancy and FBG.

Adjusted[5]: Fertility treatment, maternal age, parity.

Adjusted[6]: Income, and number of physician visits in the 3 years before the index date.

Adjusted[7]: Age, socioeconomic level, smoking status, BMI, parity, number of general practitioner visits 2 years prior to the index date.

Adjusted[8]: Age at the first observed birth, birth order at the last observed pregnancy, ethnic ancestry based on the woman's father's place of birth, social class at last observed birth, categories of education, presence of other specific complications of pregnancy in any observed birth, birth defects, low or high birth weight in one or more offspring.

Figure S1

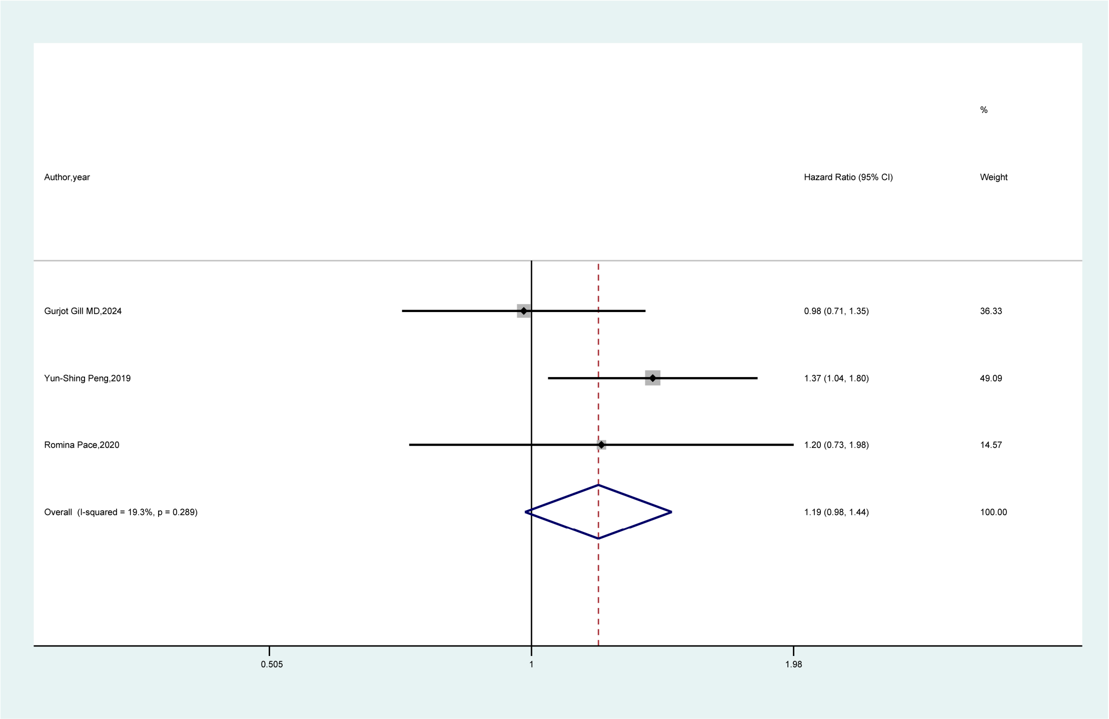

Forest plot of the association between GDM and lung cancer.

Figure S2

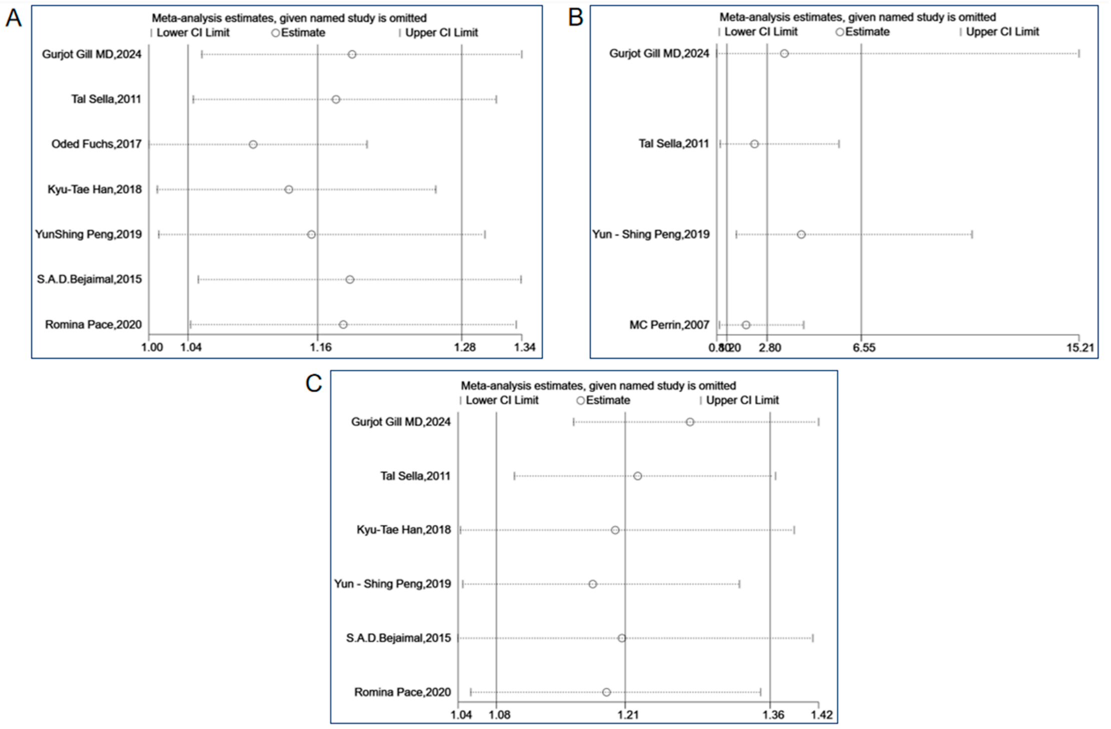

Sensitivity analyses.

A overall cancer; B pancreatic cancer; C thyroid cancer

Figure S3

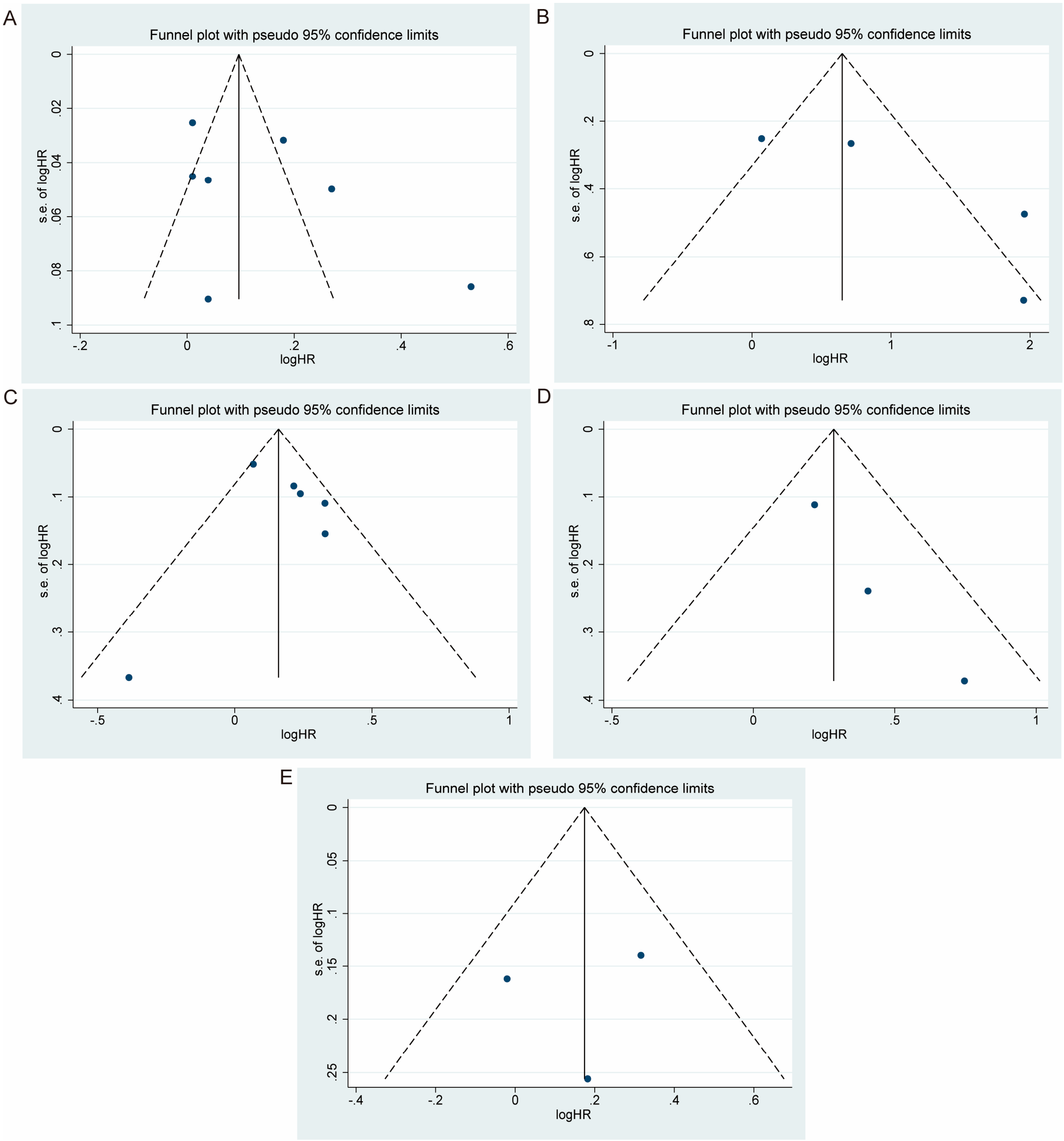

Funnel plot.

A overall cancer; B pancreatic cancer; C thyroid cancer; D liver cancer; E lung cancer

Table S3 Begg's and Egger's test values

Type of cancer	Z, P (Begg's Test)	T, P (Egger's Test)
Overall cancers	1.50, 0.333	1.25, 0.265
Pancreatic cancer	1.02, 0.308	2.24, 0.154
Thyroid cancer	0.75, 0.452	0.52, 0.628
Liver cancer	NA	NA
Lung cancer	NA	NA

NA: not applicable
